# A Three-Dimensional Finite Element Analysis of Displacement and Stress Distributions of Unilateral and Bilateral Cleft Lips by Using Developed Pre-Surgical Treatment Architecture

**DOI:** 10.3390/children8121121

**Published:** 2021-12-03

**Authors:** Ali A. H. Karah bash, Ergun Ercelebi

**Affiliations:** Department of Electrical and Electronics Engineering, University of Gaziantep, Gaziantep 27310, Turkey; ergun.ercelebi@gmail.com

**Keywords:** unilateral and bilateral cleft lip, pre-surgical treatment architecture, finite element model, mechanical and electronic parts

## Abstract

Cleft lips and cleft palates are the most common birth defects in newborns. Pre-surgical correction of unilateral and bilateral cleft lips and palates has been the subject of interest of many previous works. This condition has necessitated the evolution of many surgical and non-surgical techniques to mitigate the problem of this deformity in children. In this study, we proposed a new architecture that can be used instead of the conventional pre-surgical treatment. The proposed architecture has mechanical and electronic parts. This architecture was adopted to apply external stress to the cleft bones and cleft edges using an airbag that is located in the mechanical part. The amount of air in the airbag can be controlled by an available control unit in the electronic part. The effect of external stress on the cleft bones and the cleft edges was analyzed by using the finite element analysis (FEA) method. The FEA study aimed to analyze the displacement, amount of tensile and compressive forces, and Von Mises stress distributions on the cleft bones, cleft edges, nasal septum, and superior alveolar part of the maxillary jaw of unilateral and bilateral cleft models during pre-surgical treatment with the novel architecture. The results show that displacement and stress affected the clefts of both models. Displacement had a significant effect of gradually bringing the clefts closer to each other and returning them to the posterior. The analysis also investigated the effects of stress on the cleft bone and cleft edge. It was found from the results that the stresses helped to bring the incisions closer to the most appropriate position for plastic surgeons. The results prove that the positive and negative X-displacements move in the opposite direction, which means that the cleft edges gradually converge toward each other. Moreover, the negative Z-displacement affected the movement of cleft bones and cleft edges from outside to inside and gradually returned them to a suitable position. The findings show that the proposed architecture can be contributed to the pre-surgical treatment of the unilateral and bilateral clefts as an alternative to the traditional method.

## 1. Introduction

Cleft lips and cleft palates are the most common congenital defects worldwide. According to international studies, one out of 700 babies is born with a cleft lip, cleft palate, or both [1,2,3,4]. The current clinical practice involves monitoring and altering soft tissues in a patient [5,6,7,8,9].

A cleft can exist on the lip, in the hard or soft palate, or, less frequently, in the facial structure [10,11,12]. Clefts can be classified as complete or incomplete, and can also be unilateral or bilateral [13,14]. Furthermore, all oral fissures can be classified for lip and palate clefts. Isolated clefts in the palate are less common compared to other types of cleft [15]. Genetic mutations and environmental factors are among the most significant causes of congenital deformities that affect the fetus during the early stages of its development [12,13]. In addition, drinking alcohol and smoking can have significant effects on the occurrence of clefts [16,17]. While genes play a significant role in the development of oral-facial clefts, they are not the unique cause of these congenital deformities [18]. In the literature [19,20,21], the researchers explained that many patients have a complete cleft palate. Other patients have an incomplete cleft palate. In other cases, these fissures reach the alveolar ridges and the secondary palate.

Batwa and other researchers used several corrective devices to make preliminary corrections before performing plastic surgery, some of whom placed the devices inside the mouth, while the others used them as ligaments outside the mouth [22,23,24]. These rubber bands are attached to the cheeks to provide some external forces that contribute to the alveolar molding process.

In other articles, the researchers used surgical tape bound to an intraoral palatine plate to treat cleft lips in children [25,26,27]. The surgical tape was fastened to the cheeks of the children towards the cleft lip. The surgical tape created pressure that helped to return the cleft lip to its normal position. In recent years, finite element analysis (FEA) has established itself as a powerful research tool for solving diverse mechanical and stress analysis problems [28,29,30,31]. The researchers in Ref. [32] worked to develop the Nasoalveolar Molding (NAM) device by creating a nasal stent with a TMA wire. They used this device to reshape the nasal cartilage, maxillary arch, and cleft palate as a pre-surgery treatment. The clinical practices proved that the device was very efficient. Other researchers in Refs. [33,34] used traditional NAM with split-NAM devices in the preoperative treatment of patients with a cleft lip and palate. In the clinical studies, they showed that the split-NAM device did not work as well as the NAM device in children with a unilateral cleft lip in the transverse direction.

Zhang and other researchers analyzed the displacement and stress distribution of the maxilla and zygomatic arch using the FEA method. The research results show that there were maximum tensile and minimum compressive stresses at the zygomaticomaxillary suture areas. They used the traditional labiolingual arch in their study. From their clinical results, they concluded that the tool is suitable for skeletal patients [32]. In another study [24], the pattern of stress distribution and displacement in the middle palatal suture area was presented using a bone-borne palatal expander (BBPE) in a patient with a unilateral cleft lip and palate using FEA. The results show that the maximum displacement value was in the mid-palatal cleft area.

In addition, the researchers in Ref. [35] used the FEA method to investigate the extent of the upper craniofacial complex in patients with unilateral and bilateral cleft palates. The researchers analyzed the influence of displacement, compression, and tensile forces on unilateral and bilateral skull models. The stretching impact on the cleft side in the models used in the literature was less than on the non-cleft side.

The researchers in Ref. [36] studied jaw lengthening for patients with a cleft lip and palate. The researchers used the triple finite element model to analyze the impact of displacement and forces on maxillary expansion. They concluded that applying direct forces to the upper jaw increased the amount of displacement, which led to greater expansion in these areas.

The traditional method of pre-surgical treatment used medical tape to tighten the ends of the cleft lip. It helps to reduce the opening area of the cleft lip as much as possible. However, with the use of medical tape over a long period, the child’s cheek swells owing to the pasting and removal of the medical tape several times during the day.

The aim of this study is to build a new architecture that contains a control model and uses pressure and tension in the preoperative treatment of children with unilateral and bilateral cleft lips and palates. The amount of pressure can be controlled according to the user’s desires, and this feature is not available in the traditional methods mentioned in the previous literature. In addition, we used the FEA method for stress and force analysis to demonstrate the efficacy and efficiency of the proposed architecture by analyzing and evaluating the distributions of displacement, tensile, compressive, and von Mises stresses across the cleft bones, cleft edges, nasal septum, and superior alveolar part of the maxillary jaw using two skull models with unilateral and bilateral clefts. We used different values of the pressure provided by the proposed architecture to investigate the pressure’s effects on the cleft bones, cleft edges, nasal septum, and superior alveolar part of the maxillary jaw when the pressure values are changed. 

## 2. Materials and Methods

**Figure 1:**
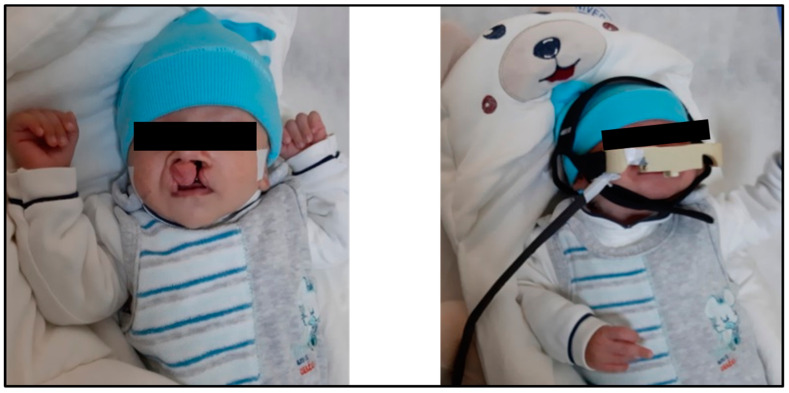
How the proposed architecture of this study is mounted on the head of a patient with a bilateral cleft lip and palate.

The protocol of this research was reviewed and approved by the scientific board of our university (Number: E-28313576-300-4516). The typical sizes of a child’s head from birth until the third month of age were retrieved from a previous study, as listed in Table 1 [37]. The circumference of the child’s head is typically measured around the largest area of the head, beginning from the top of the eyebrows towards the back of the head. When measuring the circumference of the child’s head, a non-rubber tape should be used. After that, the tape measure the broadest possible diameter of the child’s head, starting from the front area over the eyebrows to the most prominent point at the back of the head. The measurement is repeated thrice, then the highest value is recorded. In several cases, the circumference of the head of the newborn is measured on the first day after birth, and most studies that refer to the natural dimensions for the head circumference of the newborn depend on recording this value during the first 24 h of the child’s life [38]. Figure 1 shows how the proposed architecture is placed on the head of a patient with a bilateral cleft lip.

### 2.1. Child’s Skull Representation

**Figure 2:**
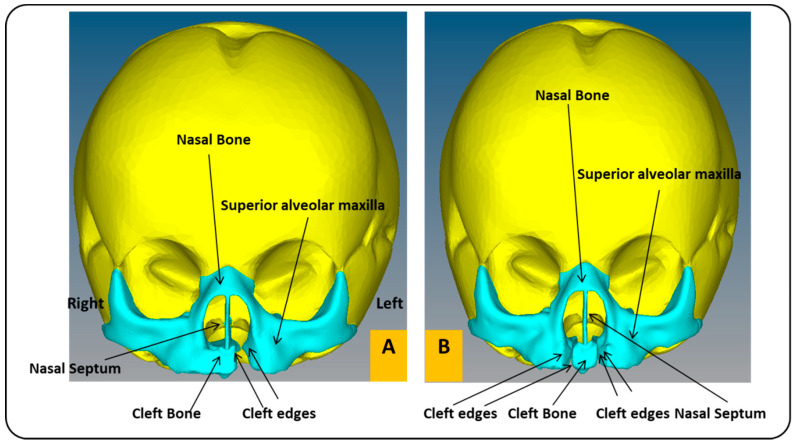
Cross-section of a child’s skull with unilateral and bilateral cleft lip and palate models: (**A**) frontal view of FEA model of child with unilateral cleft lip and palate skull. (**B**) Frontal view of FEA model of child with bilateral cleft lip and palate skull.

In this work, we utilized two models of a child’s skull (Figure 2). These medical models were purchased from the Internet [39]. The first model has a unilateral cleft lip and palate, and the other has a bilateral cleft lip and palate, in a format compatible with the analysis software used in this article (ABAQUS 6.14 software). The two models used in the study belonged to the skull of a baby in the first month. Table 1 shows the baby’s head measurements in the first three months. In this study, measurements related to the first month were utilized. The maxillary and alveolar bones were constructed to be 1 mm thick, while the rest of the skull was 5 mm thick. Maxillofacial filament width was 0.5 mm. The mechanical properties of the materials used in this work were taken from previous studies (Table 2) [29,40].

### 2.2. Mechanical Part

**Figure 3:**
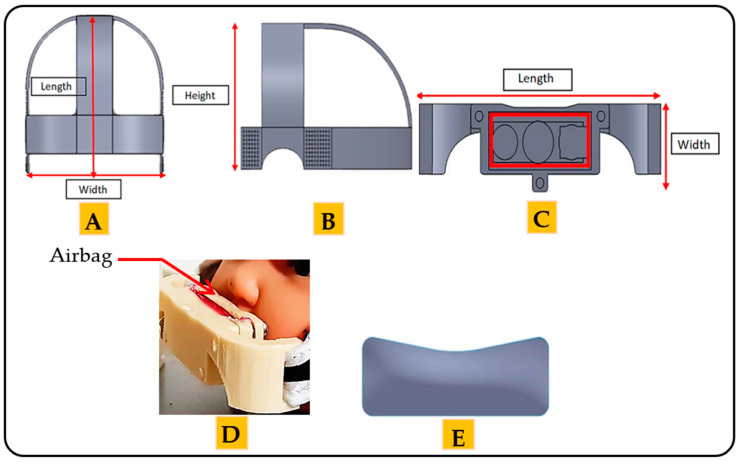
The frames of the mechanical part of the proposed architecture: (**A**) the top side of the first frame. (**B**) The lateral side of first frame. (**C**) The front view of the second frame. (**D**) Position of the airbag in the second frame. (**E**) The airbag of the proposed architecture.

The mechanical part comprises two basic frames. The function of each frame depends on the position of the frame in the architecture. The first frame looks like a hat and is placed on the head of the child. The length of the frame is 120 mm. The width is 110 mm, and the thickness is 2.5 mm (Figure 3A). The height of the frame is 67.18 mm (Figure 3B). This frame is made of a leather material; therefore, the device is suitable for human use without causing any damage to or swelling of the skin [41]. Moreover, the leather material increases the flexibility of the architecture so that the child can easily wear and remove the architecture. The mechanical properties of the leather are presented in Table 3 [28].

The second frame is considered the essential frame of the architecture. The length of the frame is 55 mm, the width is 20 mm, and the thickness is 3 mm, as shown in Figure 3C. The second frame covers the upper lip of the mouth. This frame is made of plastic, with the mechanical properties given in Table 3 [40,42].

The pressure is applied through the airbag located in the second frame (Figure 3D). The length of the airbag is 37 mm. The height is 13.46 mm, and the thickness is 2 mm. The amount of pressure can be controlled by controlling the amount of air inside the airbag. Figure 3E shows the airbag used in this architecture.

It is worth noting that the molds and frames of the mechanical part used in this article were designed with the help of an orthodontist and a healthcare professional. The mechanism of action of the proposed architecture is to provide different amounts of stress, applied directly to the cleft regions during the preoperative treatments through the airbag, since the airbag directly touches the cleft regions.

### 2.3. Electronic Part

**Figure 4:**
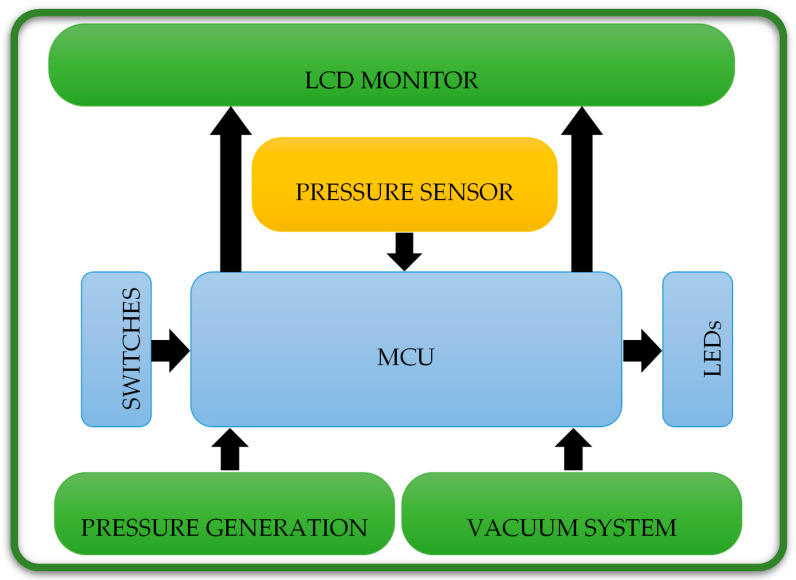
Electronic units of the electronic part of the proposed architecture in this study, which are used together with the mechanical part in the preoperative treatments.

As in Figure 4, the electronic part consists of six units, the microcontroller unit (MCU), the pressure sensor, the vacuum system, the pressure generation system, the switches and LEDs unit, and the LCD unit.

In this paper, we used a pic16f877a MCU manufactured by Microchip. This MCU is easy to program and has the characteristics of speed, flexibility, low cost, ease of use, and low consumption in many applications, such as industrial instruments, remote sensors, and safety devices. Moreover, we utilized a pressure sensor (MPXHZ6400A), which is manufactured in the USA. This sensor is applied to measure the pressure, which is between 80 and 120 KPa (600.049 and 900.074 mmHg). The small size and high reliability of this sensor make it a rational and economical choice for the system design and suitable for microcontroller-based use. This sensor is an advanced pressure sensor that provides an accurate, high-level analog output signal proportional to the pressure exerted on the sensor. The pressure can be evaluated by the transfer functions as follows:

*Vout* = *Vs* × (0.002421 X P − 0.00842) (1)

(2) $$p=\frac{Vout\;}{0.012105}+3.4779$$

where *Vout* is the output voltage of the pressure sensor, *Vs* is the input voltage (≈ 5V) and *p* is the pressure value.

**Figure 5:**
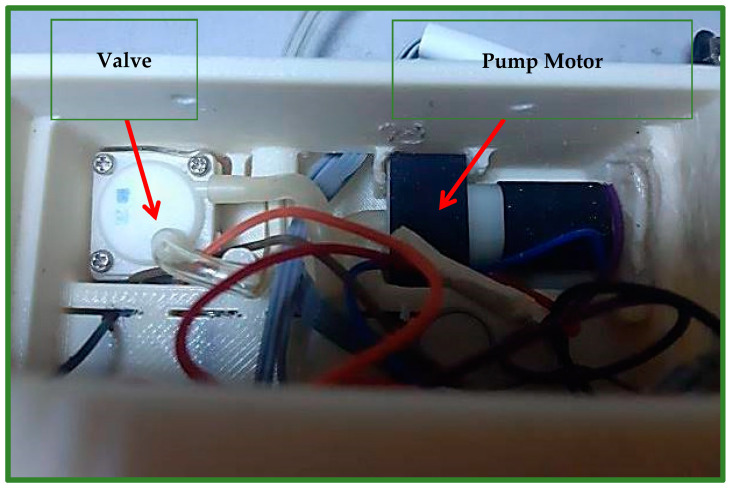
Shows the motor of the air pump, which is part of the air generation system, and the valve, which is used to deflate air, which is part of the vacuum system.

A Chinese-made SPN1501 air pump motor (Figure 5) is used to fill the airbag with a certain amount of air, so that it generates pressure on the cleft lip area. The valve (Solenoid 0520D) depicted in Figure 5 is the Chinese instrument utilized to deflate the airbag of air. The pressure will improve the locations of the cleft parts and permanently repair the lip skin and muscles [8].

## 3. Results

The study was performed using the ABAQUS 6.14 program, which has a large element library and can analyze a variety of problems. The results are represented in a band with various colors. The red color denotes areas of the highest stress or displacement. The blue color indicates areas with the least amount of stress or displacement.

### 3.1. Analysis of X-Displacement (Horizontal Direction)

**Figure 6:**
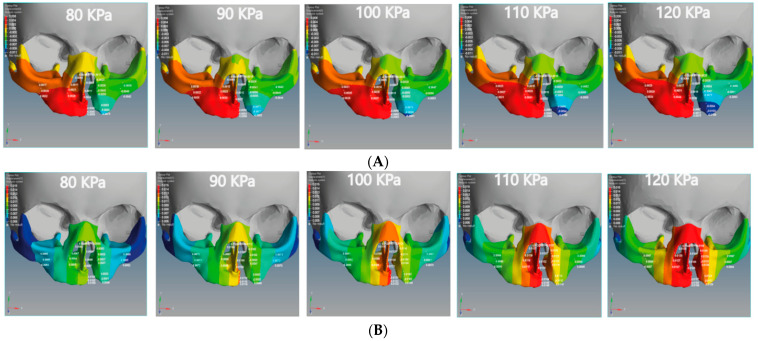

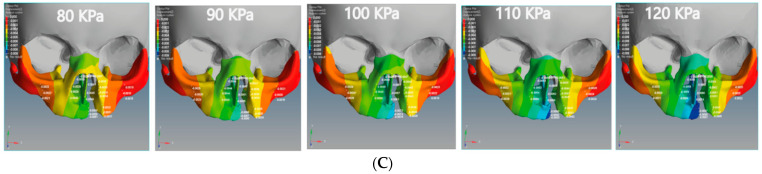
Displacement analysis of the unilateral cleft model of this study: (**A**) X-displacement analysis of the unilateral cleft model by using five different pressure values. (**B**) Y-displacement analysis of the unilateral cleft model using five different pressure values. (**C**) Z-displacement analysis of the unilateral cleft model using five different pressure values.

The negative X-displacement of the unilateral cleft model indicates the movements of the cleft bone, cleft bone edges, and left part of maxilla superiorly from the left to the right-hand side (Figure 6A).

**Figure 7:**
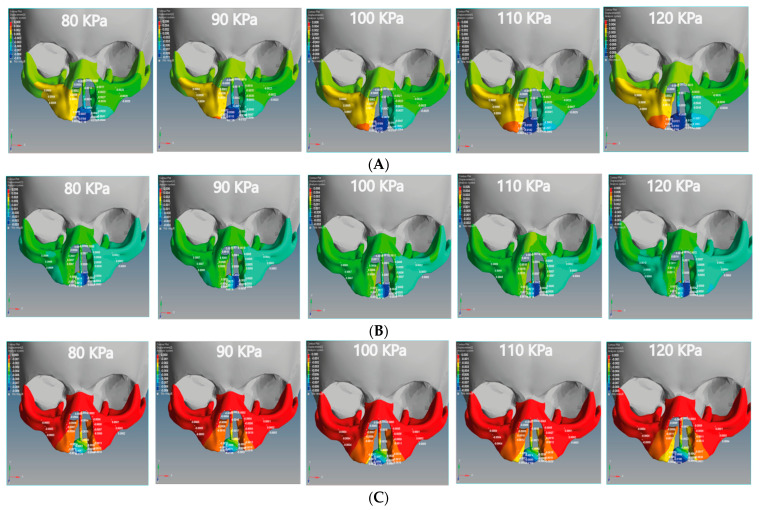
Displacement analysis of the bilateral cleft model of this study: (**A**) X-displacement analysis of the bilateral cleft model using five different pressure values. (**B**) Y-displacement analysis of the bilateral cleft model using five different pressure values. (**C**) Z-displacement analysis of the bilateral cleft model using five different pressure values.

The positive X-displacement indicates the movements of the non-cleft bone (normal site of bone), cleft bone edges, and the right part of the maxilla superior from the right to the left. As for the bilateral cleft model, the positive X-displacement refers to the right cleft bone movements, right cleft bone edges, and the right part of the maxilla superior (Figure 7A). The negative X-displacement refers to movements of the same previously mentioned areas, but on the left side.

The minimum movement values of the left and right of the maxilla superior were −0.0038 and 0.0017 mm in the unilateral cleft model and −0.0020 and 0.0004 mm in the bilateral cleft model. The maximum values were −0.006 and 0.0034 mm in the unilateral cleft model and −0.0030 mm and 0.0008 mm in the bilateral cleft model. It can also be seen that the amount of X-displacement to the right in the region of the cleft bone in the unilateral cleft model was equal to the amount on the left side. The minimum X-displacements for cleft bone and cleft bone edges were 0.0045 and −0.0063 mm, respectively. The maximum values for them were 0.0075 and −0.0109 mm for the unilateral cleft model.

Table 4 demonstrates the statistical analysis of the distribution of the displacement over the regions of the cleft bone, cleft bone edges, nasal septum, and the superior alveolar part of the maxillary jaw on the three axes. The highest amount of X displacement was on the right side in the cleft bone region. For the bilateral cleft model, the minimum X-displacement values of the cleft bone and cleft bone edges were −0.0087 mm and 0.0015 mm, respectively. The maximum values were −0.0169 and 0.0029 mm. We observed that the left and right X-displacement movements on the cleft bone edge region were equal.

### 3.2. Analysis of Y-Displacement (Up-Down Directions)

Figure 6B and Figure 7B show the effect of the upward Y-displacement for the areas of the nasal septum, superior alveolar part of the maxillary jaw, cleft bone, and cleft bone edges in the unilateral and bilateral cleft models.

We observed that the effect of the upward Y-displacement on the superior alveolar part of the maxillary jaw was more on the non-cleft side than on the cleft side. For the unilateral cleft model, the minimum values of upward Y-displacement on the cleft bone, cleft bone edges, and superior alveolar part of the maxillary jaw were 0.0105, 0.0080, and 0.0062 mm, respectively. The maximum values were 0.0159 mm, 0.0124 mm, and 0.0098 mm, respectively. In both models, the effect of Y-displacement movement was greatest in the region of the cleft bone, and it had the least effect on the left part of the maxilla superior.

Table 5 demonstrates the statistical analysis of the distributions of the displacement on the Y-coordinate (up-down directions) over the regions of the cleft bone, cleft bone edges, nasal septum, and the superior alveolar part of the maxillary jaw. The maximum values of upward Y-displacement at the cleft bone, cleft bone margins, and superior alveolar part of the maxillary jaw in the bilateral cleft model were 0.0026, 0.0013, and 0.0010 mm, respectively. The minimum values of the previously mentioned areas for the same model were −0.0028, 0.0003, and 0.0002 mm, respectively.

### 3.3. Analysis of Z-Displacement (Posterior-Anterior Directions)

The Z-displacement represents the displacement values from anterior to posterior on the *Z*-axis. Figure 6C and Figure 7C represent the Z-displacement of the unilateral and bilateral cleft models, respectively. We observed that the Z-displacement drove the superior alveolar part of the maxillary jaw to move posteriorly at the non-cleft portion more than at the cleft portion.

The minimum values of negative Z-displacement were −0.0028 and −0.0011 mm at the cleft bone and cleft bone edge in the bilateral cleft model, respectively. The maximum values for the same regions were −0.0114 and −0.0024 mm. The minimum values of posterior Z-displacement at the cleft bone and cleft bone edge were −0.0058 and −0.0031 mm in the unilateral cleft model. The maximum values were −0.0091 and −0.0047 mm.

We also observed that the superior alveolar part of the maxillary jaw movement was greater in the unilateral cleft model than in the bilateral cleft model. From the results, it can be shown that the posterior movement in the cleft edge was significantly more in the bilateral cleft model than in the unilateral cleft model.

### 3.4. The Effect of Amount of Tensile and Compressive Forces on Unilateral and Bilateral Cleft Models

**Figure 8:**
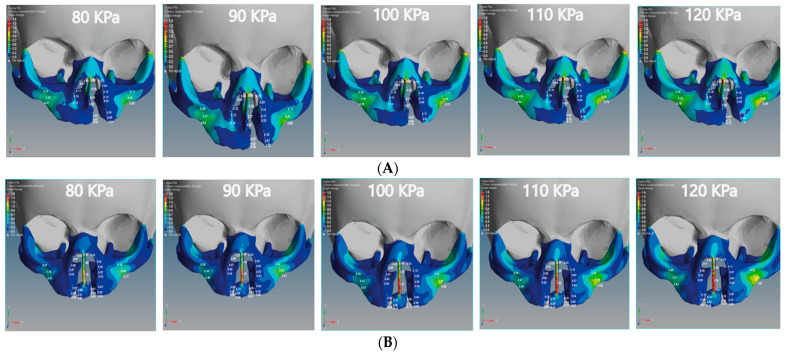
Effect of the tensile stress analysis on the regions of the cleft bone, cleft bone edges, nasal septum, and the superior alveolar part of the maxillary jaw for the unilateral and bilateral cleft models of this study: (**A**) tensile stress effects of the unilateral cleft model using different pressure values. (**B**) Tensile stress effects of the bilateral cleft model using different pressure values.

In this study, we divided the main stress into tensile and compression stresses. The positive value indicates tensile stress, and the negative value indicates compression stress. Figure 8 illustrates the tensile and compressive stresses distributions on the cleft bone, superior alveolar part of the maxillary jaw, nasal septum, and cleft bone margins in the unilateral and bilateral cleft models.

From Figure 8A, it can be seen that the tensile force affects the upward direction on the Y-coordinate for the unilateral cleft model. From Figure 8B, it can be observed that the tensile force in the bilateral cleft model affects the X-coordinate. The lowest tensile was 0.02 MPa at the nasal septum for the unilateral cleft and 0.03 MPa at the right cleft bone edge for the bilateral cleft model. The highest tensile was 0.86 MPa at the left part of the maxilla superior in the unilateral cleft and 1.76 MPa at the nasal septum in the bilateral cleft. From the results, it can be said that the tensile strength of the unilateral cleft model affects the cleft bone edge region more than the non-cleft edge region.

**Figure 9:**
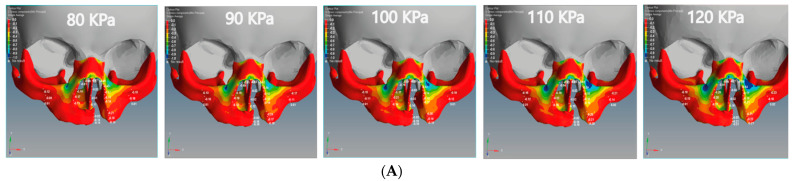

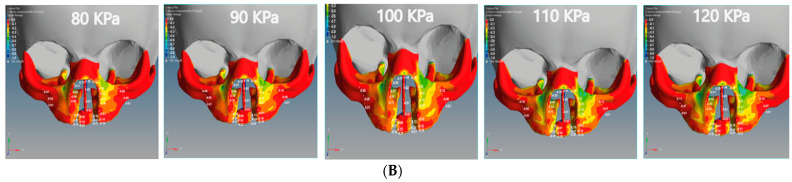
Effect of the compressive stress analysis on the regions of the cleft bone, cleft bone edges, nasal septum, and the superior alveolar part of the maxillary jaw for the unilateral and bilateral cleft models of this study: (**A**) compressive stress effects of the unilateral cleft model using different pressure values. (**B**) Compressive stress effects of the bilateral cleft model using different pressure values.

It can be seen from Figure 9A that the compressive force in the unilateral cleft model is significantly affected by the posterior direction of the Z-coordinate. In addition, the compressive force for the bilateral cleft model is influenced by the upward Y-coordinate (Figure 9B). For the unilateral cleft model, the minimum compressive force value was −0.01 MPa at the nasal septum and the highest value was −0.71 MPa in the same region.

For the bilateral cleft model, the minimum compressive force value was −0.01 MPa at the left cleft edge region and the maximum value was −0.45 MPa at the nasal septum region. Table 6 shows the statistical analysis of all tensile and compressive forces using different pressure values in the unilateral and bilateral cleft models.

### 3.5. Von Mises Stress Effects of Unilateral and Bilateral Cleft Models

**Figure 10:**
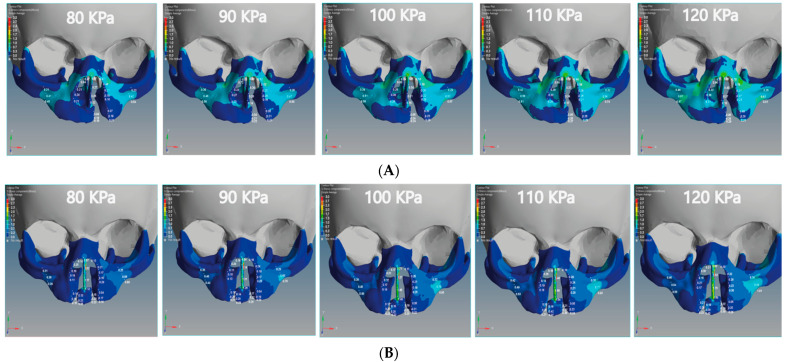
Effect of the von Mises stress analysis of the unilateral and bilateral cleft models of this study: (**A**) von Mises stress effects of the unilateral cleft model using different pressure values. (**B**) von Mises stress effects of the bilateral cleft model using different pressure values.

We analyzed the effects of von Mises stresses on the two skull models. In Figure 10A, we see that the stress is concentrated on the posterior surface on the Z-coordinate of the unilateral cleft model. The lowest value of von Mises stress for the unilateral cleft model was 0.04 MPa at the cleft bone, and the highest value was 0.74 MPa at the superior alveolar part of the maxillary jaw. For the bilateral cleft model (Figure 10B), von Mises stress concentrated on the upward surface of the Y-coordinate. The minimum stress value for the bilateral cleft model was 0.04 MPa at the cleft edge, and the maximum value was 1.75 MPa at the nasal septum. Table 7 shows all values of von Mises stress in both the unilateral and bilateral cleft model.

## 4. Discussion

According to what we have seen with the results of the FEA, the bone and gum (gingiva) under the unilateral and bilateral cleft lip and palate can be corrected and returned to their normal position by applying external pressure using the proposed architecture. As can be seen from Figure 6 and Figure 7, the displacement was asymmetrical on the cleft and non-cleft sides for both unilateral and bilateral clefts under different external forces. This result closely resembles the previous literature results, as the displacement effects for both models of unilateral and bilateral clefts were different [43].

In Table 4 and Table 5, it may be observed that the negative displacement on the left and the positive displacement on the right gradually converge in opposite directions when the external pressure is increased. As a result, the left and right edges of the cleft bone gradually converge. The present study concludes that the leftward displacement of the bilateral cleft model at the superior alveolar part of the maxillary jaw area was greater compared to that on the right side. This finding is similar to the results investigated by many finite element analysis studies [44,45].

From the results, it can be demonstrated that the cleft bone and its edges are closer to the middle in the left than on the right. Moreover, it was found that the left displacement of the unilateral cleft model had a greater effect on the cleft bone edge and cleft bone than the right displacement. Interestingly, the effect and efficiency of the X-displacement were greater in the unilateral cleft model in the cleft bone than in the bilateral cleft model. This result is in line with the results investigated by previous finite element analysis studies [46].

From Table 4 and Table 5, it can be seen that the negative X-displacement at the cleft bone edges was highest in the bilateral cleft model; this means that the ratio of the cleft bone edges’ convergence from the left side to middle was fast. We found that the right cleft bone and left cleft bone edge moved to the right. This means that the edges gradually converge towards each other under direct pressure emphasis, which is the main objective of this study.

It is noteworthy that the effect and efficacy of the upward Y-displacement in the unilateral cleft model were greater than the bilateral cleft model when using the same external pressure values (Figure 6B and Figure 7B). Additionally, we also found that the superior value of upward Y-displacement was in the cleft bone and gradually decreased when ascending to the nasal bone. We observed this case in the unilateral cleft model more often than in the bilateral cleft model. It can also be said that the Y-displacement movements on the cleft bone and the cleft bone edge were greater in the bilateral cleft model than in the unilateral cleft model. This agrees with a previous study that showed a greater Y-displacement in the bilateral cleft model than in the unilateral cleft model [45].

From the negative Z-displacement, we observed that the cleft bones and cleft bone edges of both unilateral and bilateral clefts have moved inward. Figure 6C and Figure 7C indicated that the internal Z-displacement movement of the cleft bone in the unilateral cleft was greater than that of the bilateral cleft, which means that the cleft bone and cleft bone edge in the first model moved more posteriorly than the second model. Based on the results, it can be said that the effect and efficacy of posterior Z-displacement in the bilateral cleft model surpass that of the unilateral cleft model in some areas. We also find that the effect of the Z-displacement acts directly on the bone cleft, causing it to move inward and return to the natural position, which is most favorable to the plastic surgeon. An earlier systematic review reported results close to those reported above [47]. From the results, the Z displacement effect was on the cleft bone and its edges directly, in which the cleft bones and cleft bone edges moved inward when the pressure was applied from the outside. Moreover, the movement increased with the increase in the applied external pressure.

This study noted that there are tensile and compression forces affecting the nasal septum, cleft bone, cleft bone edges, and superior alveolar part of the maxillary jaw, resulting from applying external pressure with different values by using the proposed architecture. The direction of the tensile forces was different in each of the unilateral and bilateral clefts. As can be observed from the results, the effect on the cleft bone and cleft bone edge was from below, while the effect on the nasal septum and the nasal bone was from above, and thus the effect was more upward.

It is also noteworthy that the effect of the tensile stress focused on the cleft bone and cleft bone edge in the bilateral cleft model (Figure 8B). The results also demonstrate that the effect of tensile stress was much greater in the bilateral cleft model than in the unilateral cleft model under the influence of the same value and direction of the external pressure exerted on the two models. The results show that the effect of tensile stress on the left part of maxilla superior region was more concentrated than on the right region in the unilateral and bilateral cleft models.

The results indicate that the tensile stress effect in the unilateral and bilateral cleft models on the cleft bone caused it to gradually converge on the X-coordinate with increasing external pressure, which contributed to the achievement of the aim of this study. The videos (V1, V2, V3).provided in the Supplementary Materials section can be viewed for further clarification.

Table 6 shows that the effect of compressive stress on the left cleft bone edge in the unilateral cleft model was greater than on the non-cleft bone edge. In addition, the effect of compressive stress on the left part of the maxilla superior was more concentrated than on the right side, indicating the gradual convergence of the cleft. Through the results, we can say that the effect of compressive stress on the cleft edge was more concentrated in the unilateral cleft model than in the bilateral cleft model, which helps to bring the cleft bone edges closer together (Figure 9A,B).

Based on Figure 10A,B, it can be stated that the distribution of von Mises stresses was uneven and began to affect the cleft bone and cleft bone edge, tilting back toward the negative Z-coordinate in the unilateral cleft model. As for the distribution of von Mises stress in the bilateral cleft model, it started from the cleft bone and the gum (gingiva), and then moved up toward the anterior nose and nasal septum on the positive Y-coordinate. From Table 7, the results indicate that the effect and effectiveness of von Mises stress were greater in the bilateral cleft compared to the unilateral cleft. The results also demonstrate that the effect of von Mises stress on the cleft bone and cleft bone edge regions was more in the bilateral cleft model than in the unilateral cleft model.

### Limitations

It is worth mentioning that bone regeneration takes a long time. Therefore, we could not establish a relationship between displacement and stress over time due to the difficulty of its implementation in FEA. In addition, the properties of some materials, such as skin and muscle, are unknown. We modeled the first moment when pressure is applied from the proposed architecture to the cleft areas. We would also like to mention that the external pressure exerted by the proposed architecture has specific ranges determined through the pressure sensor, ranging between 80 and 120 KPa.

## 5. Conclusions

This study proposes a new architecture to perform preoperative treatment in patients with unilateral and bilateral cleft lips and palates. This architecture consists of mechanical and electronic parts. The electronic elements are used in conjunction with mechanical frames to accomplish pre-surgical treatment for patients with unilateral and bilateral cleft lips and palates. The pressure supplied from the architecture is analyzed using the FEA method.

Based on the FEA of displacement and stress, it seemed that the cleft bone and the cleft bone edge were influenced due to the external stress exerted by the proposed architecture more significantly among the areas tested in this investigation. It is important to note that the effect of the displacement was to move the cleft bone and cleft bone edge from the outside to the inside and return them to the most suitable location for plastic surgeons.

The results show that the stresses significantly affected areas of the cleft bone and had a particular effect on the Z-coordinate, which helped to return the cleft bones and their edges to the appropriate positions. From the findings, we conclude that the proposed architecture can be used in the pre-surgical treatment due to its important role and efficiency in the pre-surgical treatment of unilateral and bilateral cleft lips and palates.

## 6. Recommendations for Future Study

The future clinical research should be planned to confirm the findings of this study.

## Figures and Tables

**Table 1:** Sizes of a child’s head at different ages. These dimensions are important in designing the frames of the proposed architecture [37].

Age	Normal Range of Head Circumference (cm)	Ideal Head Circumference (cm)
At birth	33–37	35
First month	35–38	37
Second month	37–40	39
Third month	39–41	40

**Table 2:** Values of elastic modulus, density, and Poisson’s ratio for the materials used in the skull models, which were retrieved from the previous literature. [29,38].

Material	Elastic Modulus (MPa)	Poisson Ratio	Density (kg/m^3^)
Cortical bone	13,700	0.3	1600
Cancellous bone	1370	0.3	160
Soft Tissue	0.05	0.49	925

**Table 3:** Mechanical properties of the leather and the plastic materials that are used in the frames of the mechanical part of the architecture proposed in this study. These mechanical properties were retrieved from the previous literature. [28,40,42].

Materials	Thickness (mm)	Tensile Strength (MPa)	Elongation at Break %	Young’s Modulus (MPa)
Leather	1.71 ± 0.25	21.9 ± 1.7	37 ± 3	68.8
Plastic	0.5–3	22 ± 1.5	6	1360

**Table 4:** Shows the effect of displacement distributions along the *X*-axis, *Y*-axis, and *Z*-axis (mm) on the regions of the cleft bone, cleft bone edges, nasal septum, and the superior alveolar part of the maxillary jaw for the unilateral cleft model.

Coordinates	Pressures (Kpa)	Regions							
		Cleft Bone		Cleft Bone Edges		Nasal Septum		Superior Alveolar Part of the Maxillary Jaw	
		Min.	Max.	Min.	Max.	Min.	Max.	Min.	Max.
Rightward *X*-axis (mm)	80	0.0045	0.0050	−0.0063	−0.0073	−0.0005	0.0024	0.0017	0.0022
	90	0.0050	0.0056	−0.0071	−0.0082	−0.0006	0.0027	0.0019	0.0025
	100	0.0056	0.0063	−0.0078	−0.0091	−0.0007	0.0030	0.0021	0.0028
	110	0.0061	0.0069	−0.0086	−0.0100	−0.0007	0.0033	0.0023	0.0031
	120	0.0067	0.0075	−0.0094	−0.0109	−0.0008	0.0036	0.0025	0.0034
Leftward *X*-axis (mm)	80	0.0045	0.0050	−0.0063	−0.0073	−0.0005	0.0024	−0.0038	−0.0042
	90	0.0050	0.0056	−0.0071	−0.0082	−0.0006	0.0027	−0.0042	−0.0048
	100	0.0056	0.0063	−0.0078	−0.0091	−0.0007	0.0030	−0.0047	−0.0053
	110	0.0061	0.0069	−0.0086	−0.0100	−0.0007	0.0033	−0.0052	−0.0058
	120	0.0067	0.0075	−0.0094	−0.0109	−0.0008	0.0036	−0.0056	−0.0063
Upward *Y*-axis (mm)	80	0.0105	0.0106	0.0080	0.0083	0.0096	0.0097	0.0062	0.0065
	90	0.0118	0.0119	0.0090	0.0093	0.0108	0.0109	0.0070	0.0073
	100	0.0131	0.0132	0.0100	0.0103	0.0120	0.0121	0.0078	0.0082
	110	0.0144	0.0146	0.0110	0.0114	0.0132	0.0133	0.0086	0.0090
	120	0.0157	0.0159	0.0120	0.0124	0.0144	0.0145	0.0094	0.0098
Inward *Z*-axis (mm)	80	−0.0058	−0.0061	−0.0031	−0.0032	−0.0040	−0.0049	−0.0015	−0.0023
	90	−0.0066	−0.0068	−0.0034	−0.0035	−0.0044	−0.0055	−0.0016	−0.0026
	100	−0.0073	−0.0076	−0.0038	−0.0039	−0.0049	−0.0061	−0.0018	−0.0029
	110	−0.0080	−0.0083	−0.0042	−0.0043	−0.0054	−0.0068	−0.0020	−0.0032
	120	−0.0087	−0.0091	−0.0046	−0.0047	−0.0059	−0.0074	−0.0022	−0.0035

**Table 5:** Effect of displacement distributions along the *X*-axis, *Y*-axis, and *Z*-axis (mm) on the regions of the cleft bone, cleft bone edges, nasal septum, and the superior alveolar part of the maxillary jaw for the bilateral cleft model.

Coordinates	Pressures (Kpa)	Regions							
		Cleft Bone		Cleft Bone Edges		Nasal Septum		Superior Alveolar Part of the Maxillary Jaw	
		Min.	Max.	Min.	Max.	Min.	Max.	Min.	Max.
Rightward *X*-axis (mm)	80	−0.0087	−0.0115	0.0015	0.0020	−0.0008	−0.0064	0.0004	0.0005
	90	−0.0098	−0.0130	0.0017	0.0021	−0.0009	−0.0071	0.0004	0.0006
	100	−0.0109	−0.0142	0.0019	0.0024	−0.0010	−0.0079	0.0005	0.0007
	110	−0.0120	−0.0157	0.0021	0.0027	−0.0011	−0.0087	0.0005	0.0008
	120	−0.0131	−0.0169	0.0023	0.0029	−0.0012	−0.0095	0.0006	0.0008
Leftward *X*-axis (mm)	80	−0.0081	−0.0115	−0.0038	−0.0044	−0.0008	−0.0064	−0.0020	−0.0020
	90	−0.0091	−0.0130	−0.0043	−0.0049	−0.0009	−0.0071	−0.0022	−0.0023
	100	−0.0101	−0.0142	−0.0047	−0.0054	−0.0010	−0.0079	−0.0025	−0.0025
	110	−0.0111	−0.0157	−0.0052	−0.0060	−0.0011	−0.0087	−0.0027	−0.0028
	120	−0.0121	−0.0169	−0.0057	−0.0066	−0.0012	−0.0095	−0.0030	−0.0030
Upward *Y*-axis (mm)	80	−0.0028	0.0014	0.0003	0.0009	0.0001	0.0009	0.0002	0.0006
	90	−0.0031	0.0017	0.0004	0.0010	0.0002	0.0011	0.0002	0.0007
	100	−0.0035	0.0018	0.0004	0.0018	0.0002	0.0012	0.0002	0.0008
	110	−0.0040	0.0019	0.0005	0.0012	0.0002	0.0013	0.0003	0.0009
	120	−0.0040	0.0026	0.0005	0.0013	0.0002	0.0014	0.0003	0.0010
Inward *Z*-axis (mm)	80	−0.0028	−0.0076	−0.0011	−0.0016	−0.0002	−0.0029	−0.0003	0.0002
	90	−0.0031	−0.0086	−0.0013	−0.0018	−0.0002	−0.0032	−0.0003	0.0003
	100	−0.0035	−0.0093	−0.0014	−0.0020	−0.0003	−0.0036	−0.0004	0.0003
	110	−0.0038	−0.0102	−0.0016	−0.0022	−0.0003	−0.0040	−0.0004	0.0003
	120	−0.0041	−0.0114	−0.0017	−0.0024	−0.0003	−0.0043	−0.0005	0.0004

**Table 6:** Effect of tensile and compressive stresses distributions on the regions of the cleft bone, cleft bone edges, nasal septum, and the superior alveolar part of the maxillary jaw for the unilateral and bilateral cleft models using different pressure values.

Cleft Models	Stress Type	Pressure (Kpa)	Regions							
			Cleft Bone		Cleft Bone Edges		Nasal Septum		Superior Alveolar Part of the Maxillary Jaw	
			Min.	Max.	Min.	Max.	Min.	Max.	Min.	Max.
Unilateral cleft model	Principle max (tensile) (MPa)	80	0.03	0.08	0.06	0.09	0.02	0.53	0.12	0.58
		90	0.03	0.09	0.07	0.10	0.02	0.59	0.13	0.65
		100	0.03	0.10	0.07	0.11	0.03	0.66	0.14	0.72
		110	0.04	0.11	0.08	0.12	0.03	0.73	0.16	0.79
		120	0.04	0.12	0.09	0.14	0.03	0.79	0.17	0.86
	Principle min (compressive) (MPa)	80	−0.02	−0.14	−0.14	−0.21	−0.01	−0.47	−0.15	0.01
		90	−0.02	−0.16	−0.16	−0.24	−0.02	−0.53	−0.17	0.01
		100	−0.02	−0.18	−0.18	−0.26	−0.02	−0.59	−0.19	0.01
		110	−0.02	−0.19	−0.20	−0.29	−0.02	−0.65	−0.21	0.02
		120	−0.03	−0.21	−0.21	−0.31	−0.02	−0.71	−0.23	0.02
Bilateral cleft model	Principle max (tensile) (MPa)	80	0.07	0.10	0.03	0.07	0.35	1.18	0.19	0.72
		90	0.08	0.11	0.04	0.08	0.40	1.32	0.22	0.81
		100	0.09	0.15	0.04	0.09	0.44	1.47	0.24	0.90
		110	0.13	0.20	0.04	0.09	0.49	1.62	0.26	0.99
		120	0.12	0.15	0.05	0.10	0.53	1.76	0.29	1.08
	Principle min (compressive) (MPa)	80	−0.05	−0.20	−0.01	−0.15	−0.01	−0.30	−0.09	0.01
		90	−0.06	−0.24	−0.01	−0.18	−0.01	−0.34	−0.10	0.01
		100	−0.06	−0.24	−0.02	−0.21	−0.01	−0.38	−0.12	0.01
		110	−0.07	−0.28	−0.02	−0.20	−0.01	−0.41	−0.13	0.01
		120	−0.07	−0.36	−0.02	−0.21	−0.03	−0.45	−0.14	0.01

**Table 7:** Effect of the von Mises stress distributions on the regions of the cleft bone, cleft bone edges, nasal septum, and the superior alveolar part of the maxillary jaw for the unilateral and bilateral cleft models using different pressure values.

Cleft Models	Stress Type	Pressure (Kpa)	Regions							
			Cleft Bone		Cleft Bone Edges		Nasal Septum		Superior Alveolar Part of the Maxillary Jaw	
			Min.	Max.	Min.	Max.	Min.	Max.	Min.	Max.
Von Misses (MPa)	Unilateral cleft model	80	0.04	0.19	0.19	0.27	0.12	0.87	0.23	0.54
		90	0.04	0.22	0.21	0.30	0.14	0.98	0.26	0.60
		100	0.05	0.24	0.23	0.33	0.15	1.08	0.29	0.67
		110	0.05	0.27	0.26	0.37	0.17	1.19	0.32	0.74
		120	0.06	0.29	0.28	0.40	0.18	1.30	0.35	0.81
	Bilateral cleft model	80	0.13	0.026	0.04	0.18	0.57	1.17	0.25	0.68
		90	0.14	0.29	0.04	0.21	0.64	1.31	0.28	0.76
		100	0.16	0.33	0.05	0.26	0.71	1.46	0.32	0.85
		110	0.18	0.42	0.05	0.25	0.78	1.60	0.35	0.93
		120	0.19	0.45	0.06	0.26	0.85	1.75	0.38	1.01

## Data Availability

Not applicable.

## Supplementary Materials

The following are available online at https://www.mdpi.com/article/10.3390/children8121121/s1, Video S1: Displacement Analysis Sample, Video S2: Tensile Stress Analysis Sample, Video S3: Compressive Stress Analysis Sample, Video S4: Von Mises Stress Analysis Sample.
